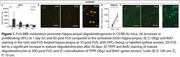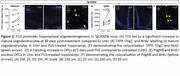# The impact of blood‐brain barrier modulation by focused ultrasound on oligodendrogenesis for Alzheimer's disease

**DOI:** 10.1002/alz70855_098200

**Published:** 2025-12-23

**Authors:** Kate Noseworthy, Joseph Silburt, Laura M Vecchio, Alessia Apa, Sheng‐Kai Wu, Luis Fernando Rubio‐Atonal, Brandy Laurette, Kullervo Hynynen, Isabelle Aubert

**Affiliations:** ^1^ Biological Sciences, Sunnybrook Research Institute, Toronto, ON, Canada; ^2^ Laboratory Medicine and Pathobiology, University of Toronto, Toronto, ON, Canada; ^3^ Sunnybrook Research Institute, Toronto, ON, Canada; ^4^ University of Toronto, Toronto, ON, Canada; ^5^ Physical Sciences, Sunnybrook Research Institute, Toronto, ON, Canada

## Abstract

**Background:**

Treatments for supporting oligodendrocytes and restoring myelin in Alzheimer disease (AD) are limited. Focused ultrasound (FUS) with microbubbles (MB) increases the permeability of the blood‐brain‐barrier (BBB) in a controlled, localized and non‐invasive manner. We have demonstrated that FUS promotes elements of brain regeneration, including hippocampal neurogenesis. To further explore the regenerative effects of FUS as a therapeutic strategy for AD, we evaluated its impact on oligodendrogenesis.

**Method:**

TgCRND8 mice were used as a model of amyloidosis. Non‐transgenic littermates and C57BL/6J mice were used as controls. FUS was unilaterally targeted to the hippocampus. Coinciding with FUS application, mice received an intravenous injection of MB; MB absorb ultrasound energy and oscillate, increasing the permeability of the BBB for 4 to 24 hours. BrdU was used to label dividing cells. In C57BL/6J mice, we quantified the proliferation of oligodendrocytes precursor cells (OPCs) at various timepoints post‐FUS to identify a time course of OPC proliferation. In all strains, oligodendrogenesis was assessed 30D post‐FUS.

**Result:**

In C57BL/6J mice (*n* = 4M per timepoint), FUS increased the number of OPCs by 6.8‐fold in FUS‐treated hippocampi compared to untreated hippocampi at 1D post‐FUS (mean±SD of 4491±2402 vs. 657±133; *p* = 0.03) and 2.3‐fold at 4D post‐FUS (2543±387 vs.1093±221; *p* = 0.002). This led to a 5.3‐fold increase in mature oligodendrocytes (882±222 vs. 166±61; *p* = 0.003) compared to the untreated hippocampiat 30D. In the presence of amyloid pathology, FUS led to a 1.4‐fold increase in mature oligodendrocytes in FUS treated compared to untreated hippocampi (317±139 vs. 231±146; *n* = 4, 2M, 2F; *p* = 0.01) at 30D post‐FUS. At 7D post‐FUS, a trending increase (*p* = 0.06) in newborn OPCs was observed in FUS‐treated (2364±1365; *n* = 4, 1M, 3F) compared to untreated hippocampi (1455±353). A sample size of 9 mice per group is estimated to reach significance with an expected medium effect size (d=0.5), alpha of 0.05 and a statistical power of 0.80.

**Conclusion:**

This first‐of‐its‐kind study demonstrates that FUS stimulates oligodendrogenesis in the mouse hippocampus, including in the context of AD pathology. Exploring the process of FUS‐induced oligodendrogenesis and gaining mechanistic insights will advance the understanding of FUS as a therapeutic modality for AD and other white matter diseases.